# Fitness of Bt‐resistant cabbage loopers on Bt cotton plants

**DOI:** 10.1111/pbi.12718

**Published:** 2017-12-20

**Authors:** Guillaume Tetreau, Ran Wang, Ping Wang

**Affiliations:** ^1^ Department of Entomology Cornell University New York State Agricultural Experiment Station Geneva NY USA; ^2^ Department of Entomology Nanjing Agricultural University Nanjing China; ^3^ Present address: IHPE UMR 5244 CNRS IFREMER Univ. Montpellier Univ. Perpignan Perpignan France; ^4^ Present address: The Institute of Plant and Environment Protection Beijing Academy of Agriculture and Forestry Sciences Beijing China

**Keywords:** fitness cost of resistance, *Trichoplusia ni*, genetically modified cotton, *Bacillus thuringiensis*, Cry1Ac, Cry2Ab

## Abstract

Development of resistance to the insecticidal toxins from *Bacillus thuringiensis* (Bt) in insects is the major threat to the continued success of transgenic Bt crops in agriculture. The fitness of Bt‐resistant insects on Bt and non‐Bt plants is a key parameter that determines the development of Bt resistance in insect populations. In this study, a comprehensive analysis of the fitness of Bt‐resistant *Trichoplusia ni* strains on Bt cotton leaves was conducted. The Bt‐resistant *T. ni* strains carried two genetically independent mechanisms of resistance to Bt toxins Cry1Ac and Cry2Ab. The effects of the two resistance mechanisms, individually and in combination, on the fitness of the *T. ni* strains on conventional non‐Bt cotton and on transgenic Bt cotton leaves expressing a single‐toxin Cry1Ac (Bollgard I) or two Bt toxins Cry1Ac and Cry2Ab (Bollgard II) were examined. The presence of Bt toxins in plants reduced the fitness of resistant insects, indicated by decreased net reproductive rate (*R*_0_) and intrinsic rate of increase (*r*). The reduction in fitness in resistant *T. ni* on Bollgard II leaves was greater than that on Bollgard I leaves. A 12.4‐day asynchrony of adult emergence between the susceptible *T. ni* grown on non‐Bt cotton leaves and the dual‐toxin‐resistant *T. ni* on Bollgard II leaves was observed. Therefore, multitoxin Bt plants not only reduce the probability for *T. ni* to develop resistance but also strongly reduce the fitness of resistant insects feeding on the plants.

## Introduction

Biotech crops engineered to express *Bacillus thuringiensis* (Bt) toxins have been widely used for insect pest control in agriculture since 1996 (James, 2015). In the past two decades, Bt crops have produced significant environmental and economic benefits (Carpenter, 2010; Cattaneo *et al*., 2006; Hutchison *et al*., 2010; Lu *et al*., 2012). However, development of resistance to Bt toxins in insects threatens the sustainable use of Bt crops (Tabashnik *et al*., 2008). Since the wide adoption of Bt crops, cases of resistance to Bt crops have been reported in several insect pests (Tabashnik and Carriere, 2015). Studies of Bt‐resistant insects have indicated that resistance is often associated with fitness cost when insects are feeding on non‐Bt host plants (Gassmann *et al*., 2009; Wang *et al*., 2016). The fitness of Bt‐resistant insects on host plants, particularly on Bt plants, is a critically important factor determining the speed of resistance development in insect populations (Carriere *et al*., 2012; Gassmann *et al*., 2009; Lenormand and Raymond, 1998; Raymond *et al*., 2005, 2007; Williams *et al*., 2011).

For delaying the development of insect resistance to Bt crops, a biotechnological strategy currently used is to engineer Bt crops to produce two or more Bt toxins to provide ‘redundant killing’ of target insect pests, known as the ‘pyramid strategy’ (Brévault *et al*., 2013; Carriere *et al*., 2015; Tabashnik *et al*., 2008; Zhao *et al*., 2003). The widely adopted resistance management strategy in the field is to preserve susceptible alleles in pest populations by planting non‐Bt crops as refuge when Bt crops are planted, known as the ‘refuge strategy’ (Gould, 1998; Tabashnik, 2008). Since 2004, pyramided Bt crops have been widely adopted and have now become the majority of transgenic Bt crops cultivated in the USA, Australia and India (Tabashnik *et al*., 2013). Although no resistance to pyramided Bt crops has been reported in the field, high level of insect resistance to two Bt toxins has been selected in the laboratory and the resistant insects can survive on the dual Bt toxin plants (Kain *et al*., 2015). In the field where both Bt and non‐Bt host plants are present, the reduced performance of resistant insects on non‐Bt plants (known as fitness cost) and on Bt crops (known as incomplete resistance) affects the selection of resistant alleles in insect populations (Garcia *et al*., 2015; Gassmann *et al*., 2009). The fitness of insects on plants can be affected by various factors, including the insect genetic backgrounds, the specific gene mutations that confer resistance to Bt toxins and the host plants that the insects feed on, in addition to the Bt toxins in the plants (Carriere *et al*., 2004, 2005; Raymond *et al*., 2005, 2007, 2011). Therefore, the fitness of Bt‐resistant insects on Bt crops impacts the efficacy of the toxin‐pyramiding and refuge strategies.

The cabbage looper, *Trichoplusia ni*, is a generalist insect herbivore with a highly diverse range of host plants, accounting for 160 plant species from 36 different families (Sutherland and Greene, 1984). *T. ni* has also developed resistance to Bt toxins in an agricultural environment (Janmaat and Myers, 2003). From the Bt‐resistant *T. ni* population, two genetically independent resistance traits, Cry1Ac resistance and Cry2Ab resistance, have been isolated and introgressed into an inbred laboratory *T. ni* strain (Song *et al*., 2015; Wang *et al*., 2007). The Cry1Ac‐resistant *T. ni* can survive on broccoli and cotton plants engineered to produce the toxin Cry1Ac (Kain *et al*., 2015; Wang *et al*., 2007), and the resistance has been mapped to an *ABCC* gene locus (Baxter *et al*., 2011), a Cry1Ac resistance gene locus known in several other lepidopterans (Baxter *et al*., 2011; Gahan *et al*., 2010; Heckel, 2012). The resistance of *T. ni* to Cry2Ab is a monogenic trait independent of Cry1Ac resistance (Song *et al*., 2015). The combination of the Cry1Ac and Cry2Ab resistance mechanisms in *T. ni* enables the insect to survive on the pyramided Bt cotton plants producing both Cry1Ac and Cry2Ab (Kain *et al*., 2015; Song *et al*., 2015). A recent study on Bt resistance in *T. ni* examined the importance of two major factors that affect the fitness cost in Bt‐resistant *T. ni* – the host plants and the specific mechanisms of resistance (Wang *et al*., 2016). They found that the insects feeding on secondary host plants (i.e. host plants other than their preferred primary host plants) showed a greater fitness cost with higher mortality, slower development and lower fecundity (Wang *et al*., 2016). Similar observations have also been reported in other Bt‐resistant lepidopteran pests on non‐Bt crop plants (Bird and Akhurst, 2007; Carriere *et al*., 2005; Janmaat and Myers, 2007; Raymond *et al*., 2007). Moreover, fitness cost associated with the resistance to Cry2Ab in *T. ni* was higher than the fitness cost associated with the resistance to Cry1Ac on nontransgenic plants (Wang *et al*., 2016).

Most studies investigating fitness costs associated with Bt resistance have been conducted on nontransgenic plants (i.e. without exposure of the insects to Bt toxins; Gassmann *et al*., 2009; Wang *et al*., 2016). Although there have been several studies reporting the fitness of insects on Bt crops producing a single toxin (Bird and Akhurst, 2004, 2005; Mahon and Olsen, 2009a,b), no studies have been reported on fitness of multitoxin‐resistant insects on Bt crops producing multiple Cry toxins. In this study, the reduction in fitness associated with resistance to a single Bt toxin and dual Bt toxins was determined using near‐isogenic *T. ni* strains grown on leaves of three cotton varieties: conventional non‐Bt cotton, the single‐toxin (Cry1Ac) Bt cotton Bollgard I and the dual‐toxin (Cry1Ac and Cry2Ab) Bt cotton Bollgard II. The near‐isogenic susceptible and Bt‐resistant *T. ni* strains and the commercial Bt cotton varieties with a similar genetic background provided a unique insect‐Bt plant system to analyse the fitness of resistant and susceptible insects on Bt crop plants and their impact on insect resistance development and resistance management.

## Results

### Population growth rate of *T. ni* decreased when the number of toxins in plants increased

**Figure 1:**
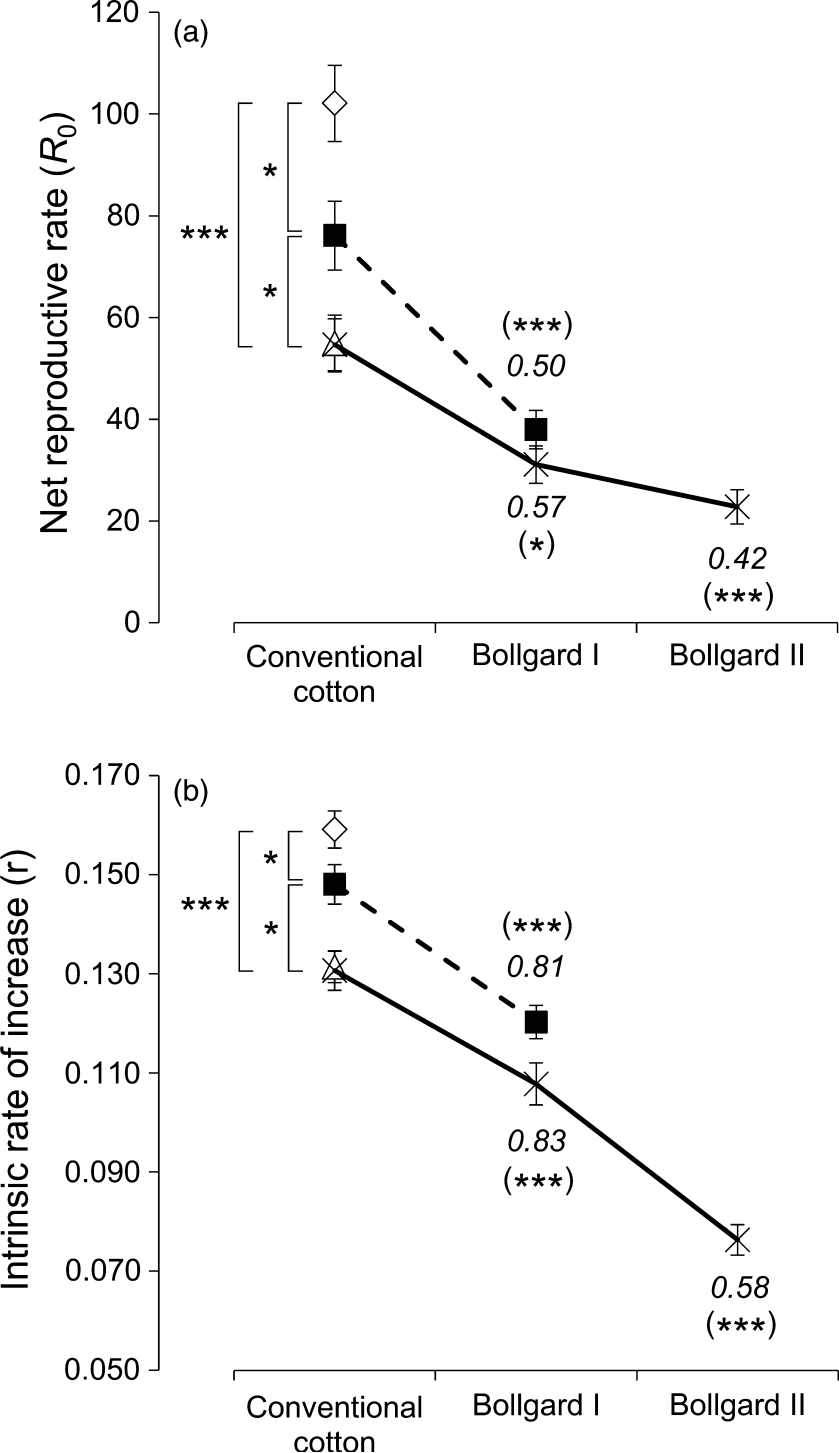
Growth of *T. ni* populations on Bt and non‐Bt cotton leaves. (a) Net reproductive rate (*R*_0_) and (b) intrinsic rate of increase (*r*) of *T. ni* populations of the susceptible strain (diamond), the Cry1Ac‐resistant strain GLEN‐Cry1Ac‐BCS (black square, dashed line), the Cry2Ab‐resistant strain GLEN‐Cry2Ab‐BCS (triangle) and the dual‐toxin‐resistant strain Cry2Ab (GLEN‐Cry1Ac+Cry2Ab‐BCS (black cross, solid line) on conventional non‐Bt cotton leaves and Bollgard I and Bollgard II cotton leaves. Error bars represent the standard errors of means for the *R*_0_ or *r* values. The statistical significance levels of the difference in population growth among the 3 *T. ni* strains on conventional cotton are indicated with the *P* values without brackets. The statistical significance levels of the difference of the same strains between feeding on transgenic plants (Bollgard I or Bollgard II) and feeding on conventional cotton plants are indicated with *P* values in brackets. The *P* values are calculated based on one‐way ANOVA and they are indicated by ‘*’ (*P *< 0.05) and ‘***’ (*P *< 0.001). Relative fitness (*w*) is the ratio of *R*_0_ or *r* of a resistant strain on Bt cotton leaves to the *R*_0_ or *r* of the same strain on non‐Bt cotton leaves and it is indicated in italic.

The Bt‐susceptible Cornell strain (Kain *et al*., 2015) and the three near‐isogenic *T. ni* strains resistant to Cry1Ac [strain GLEN‐Cry1Ac‐BCS (Wang *et al*., 2007)], to Cry2Ab [strain GLEN‐Cry2Ab‐BCS (Song *et al*., 2015)] and to both Cry1Ac and Cry2Ab [strain GLEN‐Cry1Ac+Cry2Ab‐BCS (Kain *et al*., 2015)] all survived and completed their life cycles on leaves of the conventional non‐Bt cotton plants. The GLEN‐Cry1Ac‐BCS and GLEN‐Cry1Ac+Cry2Ab‐BCS strains could survive on Bollgard I leaves, which contain Cry1Ac toxin, while GLEN‐Cry1Ac+Cry2Ab‐BCS could survive on Bollgard II leaves, which contain both Cry1Ac and Cry2Ab toxins. The net reproductive rate (NRR) *R*_0_ was above the critical value of 1 (ranging from 22.8 to 102.1) on all the cotton plants tested for the *T. ni* strains that survived on the respective cotton plants (Figure 1a). Similarly, the intrinsic rate of increase (IRI) *r* was also above the critical value 0 (ranging from 0.076 to 0.159) on all the host plants tested for the *T. ni* strains that survived on them (Figure 1b). Both the *R*_0_ and *r* values indicated that, for the *T. ni* strains that could survive on the specific cotton plants, the strains could not only survive but their populations could also grow on the Bt cotton plants. The *R*_0_ and *r* of the susceptible strain were significantly greater than those of the resistant strains on non‐Bt cotton leaves (Figure 1), indicating the fitness cost associated with the resistant traits in the *T. ni* strains fed on cotton leaves. On the non‐Bt cotton leaves, a greater decrease in *R*_0_ was observed for the *T. ni* strains that carried the Cry2Ab‐resistant mutation (from *R*_0_ = 102.1 for the susceptible strain to *R*_0_ = 54.9 and 54.6 for GLEN‐Cry2Ab‐BCS and GLEN‐Cry1Ac+Cry2Ab‐BCS strains, respectively) than that observed in the strain resistant to only Cry1Ac (*R*_0_ = 76.1 for GLEN‐Cry1Ac‐BCS; Figure 1a). The fitness of the *T. ni* strains on non‐Bt cotton and Bt cotton plants evaluated by IRI showed a similar pattern as by NRR (Figure 1b).

The Cry1Ac‐resistant strains, GLEN‐Cry1Ac‐BCS and GLEN‐Cry1Ac+Cry2Ab‐BCS, could survive on Bollgard I leaves, but the NRR was significantly decreased (Figure 1a), indicating incomplete resistance to the Bt cotton leaves. Grown on non‐Bt cotton leaves, the GLEN‐Cry1Ac+Cry2Ab‐BCS strain showed a significantly lower fitness than the GLEN‐Cry1Ac‐BCS (*P* = 0.038, one‐way ANOVA), but the two strains showed very similar relative fitness (0.50 for GLEN‐Cry1Ac‐BCS and 0.57 for GLEN‐Cry1Ac+Cry2Ab‐BCS) when grown on Bollgard I leaves as compared to same strains on non‐Bt cotton (*P *= 0.97, one‐way ANOVA; Figure 1a). This reduced fitness of the GLEN‐Cry1Ac‐BCS and GLEN‐Cry1Ac+Cry2Ab‐BCS strains on Bollgard I was similarly shown when using IRI (Figure 1b). On Bollgard II leaves with two toxins, the NRR and IRI for the GLEN‐Cry1Ac+Cry2Ab‐BCS were more greatly reduced with the relative fitness decreased further to 0.42 for NRR (*P *= 0.0009, one‐way ANOVA; Figure 1a) and to 0.58 for IRI (*P *= 0.0007, one‐way ANOVA; Figure 1b). Overall, the fitness of *T. ni* on Bt cotton leaves decreased in comparison with that on non‐Bt cotton leaves and further decreased with the increase in the number of toxins from 1 to 2 in the cotton leaves (Figure 1).

### Resistant *T. ni* strains on Bt cotton leaves had an increased mortality

**Figure 2:**
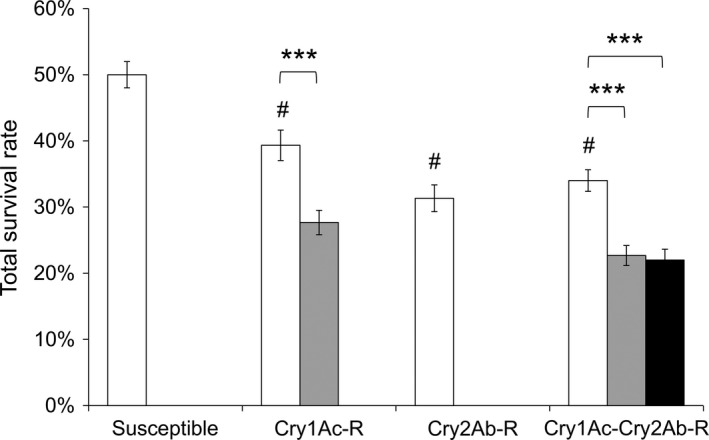
Survival of the susceptible, the GLEN‐Cry1Ac‐BCS, the GLEN‐Cry2Ab‐BCS and the GLEN‐Cry1Ac+Cry2Ab‐BCS strains on conventional cotton (white), Bollgard I (grey) and Bollgard II (black). The survival rates of the resistant strains on conventional cotton are all statistically different from that of the susceptible strain (*P *< 0.001 by one‐way ANOVA) and is indicated by a ‘#’ on top of the columns for each resistant strain. For each strain, the statistical significance level for difference in survival between the larvae on Bt and non‐Bt cotton leaves is indicated by ‘***’ (*P *< 0.001 by one‐way ANOVA). The rates of survival are represented as mean ± SE.

**Figure 3:**
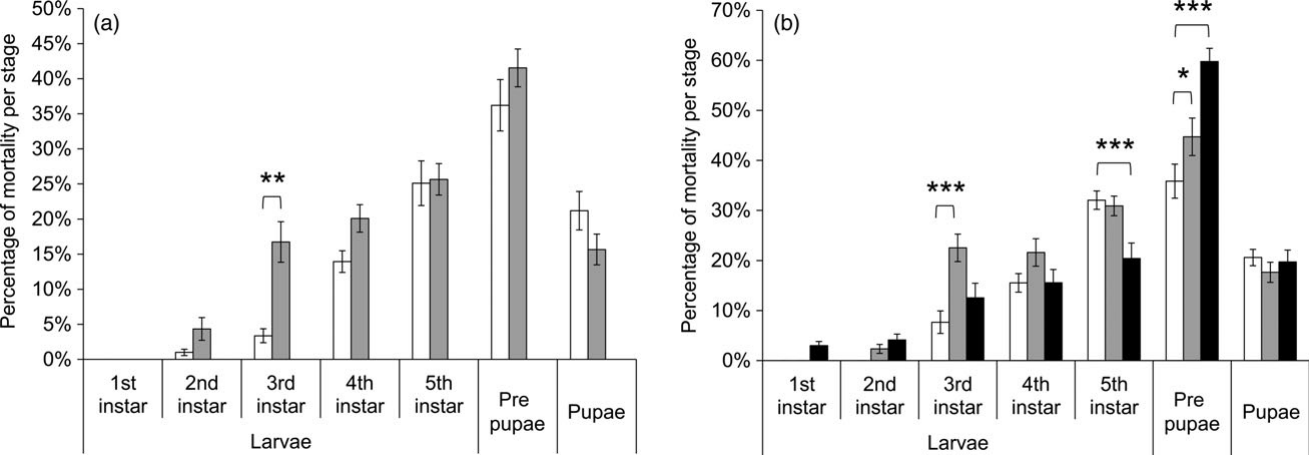
Mortality at different developmental stages of *T. ni* from (a) GLEN‐Cry1Ac‐BCS and (b) GLEN‐Cry1Ac+Cry2Ab‐BCS on leaves of conventional cotton (white), Bollgard I (grey) and Bollgard II (black). For each strain, the significance levels of difference (by one‐way ANOVA) between the mortality of *T. ni* on Bollgard I and Bollgard II plant leaves and that on conventional cotton leaves are indicated by ‘*’ (*P *< 0.05), ‘**’ (*P *< 0.01) and ‘***’ (*P *< 0.001). Values of mortality are represented as mean ± SE.

The three resistant strains exhibited a decrease of 7.7%–13% in survival rate on leaves of non‐Bt cotton as compared to the susceptible strain (Figure 2). A significant 33%–53% decrease in survival was observed for GLEN‐Cry1Ac‐BCS and GLEN‐Cry1Ac+Cry2Ab‐BCS strains on Bt cotton leaves, as compared to their survival on the non‐Bt cotton leaves (Figure 2). Larval mortality was increased both on Bollgard I and on Bollgard II plant leaves in the resistant strains in early instars. Particularly, a significantly increased mortality was observed in the 3rd instar for both GLEN‐Cry1Ac‐BCS and GLEN‐Cry1Ac+Cry2Ab‐BCS strains on Bollgard I (Figure 3). For the GLEN‐Cry1Ac+Cry2Ab‐BCS strain, the larvae on Bollgard II leaves started to show mortality in neonates and the mortality increased with the increasing instar. In contrast, larval mortality was not observed till 3rd instar when the larvae were fed on non‐Bt cotton leaves. Even though its mortality within the larval stage of 5th instar was relatively lower on Bollgard II leaves, the mortality increased significantly in the immediate subsequent stage (prepupal stage; Figure 3). Cumulatively, the larvae on Bollgard I and Bollgard II leaves had higher mortality, compared to the same strain on non‐Bt cotton leaves (Figure 2). Mortality was not affected by the sex of *T. ni*, as the sex ratio of survivors did not vary significantly on cotton leaves with or without Bt toxins (Table 1).

**Table 1:** Fitness parameters of the Bt‐susceptible and Bt‐resistant *T. ni* strains not significantly affected by the presence of toxins in plant leaves

Trait	Strain	Conventional cotton	Bollgard I	Bollgard II
		Mean ± SE (n)	Mean ± SE (n)	Mean ± SE (n)
Sex ratio (% of females)	Cornell Bt‐Susceptible	49.8 ± 3.5 (30)		
	GLEN‐Cry1Ac‐BCS	53.2 ± 5.1 (30)	47.0 ± 6.4 (30)	
	GLEN‐Cry2Ab‐BCS	51.0 ± 5.3 (30)		
	GLEN‐Cry1Ac+Cry2Ab‐BCS	48.3 ± 4.8 (30)	38.8 ± 6.0 (30)	41.5 ± 6.9 (30)
Pupal weight (mg)	Cornell Bt‐Susceptible	170.2 ± 4.8 (90)		
	GLEN‐Cry1Ac‐BCS	168.9 ± 5.7 (90)	166.6 ± 5.8 (83)	
	GLEN‐Cry2Ab‐BCS	164.8 ± 5.0 (90)		
	GLEN‐Cry1Ac+Cry2Ab‐BCS	159.7 ± 4.9 (90)	164.6 ± 5.4 (68)	158.2 ± 4.4 (66)
Emergence of healthy adults (%)	Cornell Bt‐Susceptible	78.9 ± 2.9 (30)		
	GLEN‐Cry1Ac‐BCS	77.7 ± 4.7 (30)	79.7 ± 6.0 (30)	
	GLEN‐Cry2Ab‐BCS	79.9 ± 5.3 (30)		
	GLEN‐Cry1Ac+Cry2Ab‐BCS	79.3 ± 3.5 (30)	75.6 ± 6.5 (30)	75.8 ± 6.6 (30)
Fecundity (eggs per female)	Cornell Bt‐Susceptible	638.2 ± 36.3 (20)		
	GLEN‐Cry1Ac‐BCS	588.9 ± 42.5 (17)	494.2 ± 44.2 (15)	
	GLEN‐Cry2Ab‐BCS	556.7 ± 47.9 (15)		
	GLEN‐Cry1Ac+Cry2Ab‐BCS	535.9 ± 46.2 (16)	528.7 ± 54.3 (13)	449.4 ± 50.3 (11)
Hatchability (%)	Cornell Bt‐susceptible	80.2 ± 1.2 (20)		
	GLEN‐Cry1Ac‐BCS	78.2 ± 1.7 (17)	74.2 ± 2.1 (15)	
	GLEN‐Cry2Ab‐BCS	74.8 ± 1.7 (15)		
	GLEN‐Cry1Ac+Cry2Ab‐BCS	75.1 ± 2.0 (16)	76.3 ± 2.4 (13)	72.9 ± 2.4 (11)

For all the five fitness parameters shown in this table, no significant effect of the presence of toxins in plant leaves and of the mechanism of resistance developed was observed (one‐way ANOVA; *P* > 0.05). Values are indicated as mean ± SE and the number of replicates (*n*) is indicated in brackets for each strain on each plant.

### Resistant *T. ni* developed more slowly when feeding on cotton leaves with Bt toxins

**Figure 4:**
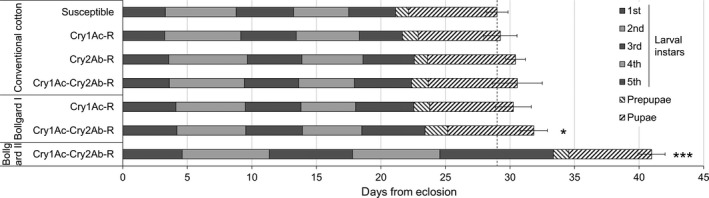
Development durations of the susceptible, GLEN‐Cry1Ac‐BCS (Cry1Ac‐R), GLEN‐Cry2AB‐BCS (Cry2Ab‐R) and GLEN‐Cry1Ac+Cry2Ab‐BCS (Cry1Ac‐Cry2Ab‐R) strains on non‐Bt (conventional), Bollgard I and Bollgard II cottons. Average development durations of larval instars are indicated in alternate solid dark and light grey, and those for prepupal and pupal stages are indicated with hatched lines. A vertical dashed line indicates the total development duration of susceptible individuals on conventional cotton from neonates to adult emergence. Significant difference (by one‐way ANOVA) of the total development duration, from egg hatching to adult emergence, in the same *T. ni* strain when feeding Bollgard I and Bollgard II, compared to feeding on non‐Bt cotton leaves is indicated by a ‘*’ (*P *< 0.05) or ‘***’ (*P *< 0.001). Values are represented as mean ± SE.

When reared on non‐Bt cotton leaves, the susceptible and the three resistant strains of *T. ni* developed from neonates to adult emergence in 22.2–23.7 days on average without statistically significant difference among the *T. ni* strains (Figure 4). However, the developmental duration of the GLEN‐Cry1Ac+Cry2Ab‐BCS strain was 1.5 days longer on Bollgard I leaves (one‐way ANOVA, *P *= 0.019) and 10.9 days longer on Bollgard II leaves (*P *= 0.0001), compared with its development time on non‐Bt cotton leaves (Figure 4). The GLEN‐Cry1Ac+Cry2Ab‐BCS strain grown on Bollgard II leaves reached the adult stage 12.4 days later than the susceptible strain grown on non‐Bt cotton leaves (Figure 4).

**Figure 5:**
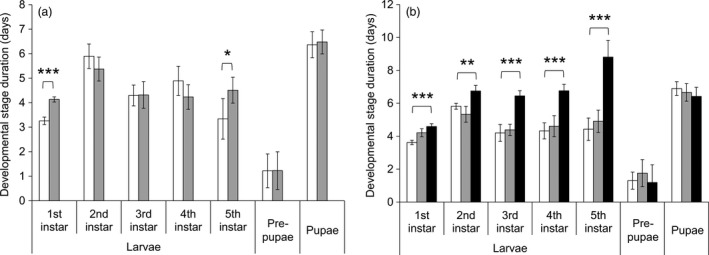
Durations of individual developmental stages of (a) GLEN‐Cry1Ac‐BCS and (b) GLEN‐Cry1Ac+Cry2Ab‐BCS on leaves from conventional cotton (white), Bollgard I (grey) and Bollgard II (black) plants. For each strains, the statistical significance of difference between the *T. ni* on non‐Bt cotton leaves and on leaves from Bollgard I and Bollgard II plants were analysed by one‐way ANOVA, and the level of statistical significance of the differences are indicated by ‘*’ (*P *< 0.05), ‘**’ (*P *< 0.01) and ‘***’ (*P *< 0.001). Values are represented as mean ± SE.

The effect of Bt cotton leaves on the duration of larval development varied, depending on the instars, the toxins in plants and the specific resistance mechanisms in *T. ni*. In the Cry1Ac‐resistant GLEN‐Cry1Ac‐BCS strain, the duration of larval development was not significantly slowed on Bollgard I (Figure 5a). In the dual‐toxin‐resistant GLEN‐Cry1Ac+Cry2Ab‐BCS strain, the larval development durations were not different in all instars between feeding on non‐Bt and Bollgard I cotton leaves (Figure 5b), which is similar to what was observed in GLEN‐Cry1Ac‐BCS larvae on Bollgard I leaves (Figure 5a). However, the larval developmental duration of the GLEN‐Cry1Ac+Cry2Ab‐BCS strain was significantly increased at all larval instars when feeding on Bollgard II, as compared to feeding on non‐Bt cotton (Figure 5b). In spite of the delayed larval development on Bollgard II leaves, no significant difference in the durations of prepupal nor in pupal stages was observed between those feeding on non‐Bt and on Bt cotton leaves in the GLEN‐Cry1Ac+Cry2Ab‐BCS strain (Figure 4). The extended development time of *T. ni* on Bt cotton leaves did not result in a significant change of pupal weight nor in the rate of emergence of healthy adults (Table 1).

### Feeding on dual‐toxin cotton (Bollgard II) leaves affected the kinetics of egg laying in *T. ni*

**Figure 6:**
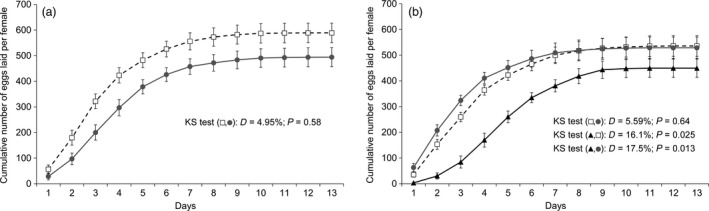
Kinetics of egg laying of females from (a) GLEN‐Cry1Ac‐BCS and (b) GLEN‐Cry1Ac+Cry2Ab‐BCS strains reared on conventional cotton (white squares, discontinuous line), on Bollgard I (grey circles and line) and on Bollgard II (black triangles and line). Numbers of cumulative egg laying are indicated as mean ± SE. Values of the Kolmogorov–Smirnov test for each pairwise comparisons performed are indicated.

Feeding on Bollgard I leaves did not significantly affect the egg‐laying kinetics of both GLEN‐Cry1Ac‐BCS (two‐sample two‐sided Kolmogorov–Smirnov analysis, *D* = 4.98%, *P *= 0.58; Figure 6a) and GLEN‐Cry1Ac+Cry2Ab‐BCS (*D* = 5.59%, *P *= 0.64) strains (Figure 6b), as compared to these strains feeding on non‐Bt cotton leaves. However, significantly different patterns of egg laying were observed in the GLEN‐Cry1Ac+Cry2Ab‐BCS strain when reared on Bollgard II leaves as compared to the strain on conventional cotton (*D* = 16.1%, *P *= 0.025) and to Bollgard I leaves (*D* = 17.5%, *P *= 0.013; Figure 6b). The significantly different egg‐laying pattern of the GLEN‐Cry1Ac+Cry2Ab‐BCS strain reared on Bollgard II leaves showed slower egg laying of this strain on Bollgard II plant leaves.

The total number of eggs laid per female and the rate of egg hatching on average were lower in *T. ni* fed with leaves from Bt cotton plants than those on non‐Bt plants (Table 1). However, the decrease in both the total number of eggs laid and the rate of egg hatching was not statistically significant (Table 1).

## Discussion

Bt resistance in insects is often associated with a fitness cost, and the level of fitness reduction is affected by the specific resistance‐conferring mutations and also by physiological stresses that the insects encounter from feeding on host plants with different nutritional factors and various allelochemicals (Bird and Akhurst, 2004, 2005; Janmaat and Myers, 2005, 2007; Mahon and Olsen, 2009a; Raymond *et al*., 2005, 2007, 2011; Wang *et al*., 2016; Williams *et al*., 2011). The Bt‐resistant *T. ni* strains exhibit a higher fitness cost when feeding on an alternative host plant, comparing with feeding on well‐adapted preferred host plants (Janmaat and Myers, 2005, 2007; Wang *et al*., 2016). When feeding on Bt plants, the insects experience physiological stress from the toxicity of Bt toxins, even though the insects may have developed resistance to the Bt toxins, and also from plant allelochemicals. The unique Bt‐susceptible and resistant near‐isogenic strains of *T. ni* provided an opportunity for analysis of the effects of Bt toxins Cry1Ac and Cry2Ab in transgenic cotton plants on the fitness of Bt‐resistant *T. ni* strains with two specific genetically independent Bt resistance mechanisms.

Based on the net reproductive rate *R*_0_ and the intrinsic rate of increase *r* of the *T. ni* strains grown on non‐Bt cotton leaves, all Bt‐resistant *T. ni* strains showed reduced fitness on cotton leaves. In other words, there is a fitness cost associated with Bt resistance in the *T. ni* strains feeding on non‐Bt cotton plants, as previously reported by Wang *et al*. (2016). Between the *T. ni* strains with the Cry1Ac and Cry2Ab resistance, the fitness of Cry2Ab‐resistant individuals was lower than that of the Cry1Ac‐resistant individuals. This is consistent with the previous report in which the very same parameters were also found lower in Cry2Ab‐resistant strains when *T. ni* was reared on artificial diet, cabbage, tobacco and tomato leaves (Wang *et al*., 2016). The presence of a single toxin in cotton plants (i.e. Cry1Ac in Bollgard I) further reduced the fitness of the Cry1Ac‐resistant *T. ni* strains when grown on Bollgard I leaves by a moderate decrease in larval survival and rate of larval development. The relative fitness of Cry1Ac‐resistant *T. ni* on Bollgard I determined in this study (0.50–0.57 by *R*_0_ and 0.81–0.83 by *r*, see Figure 1) was higher than the relative fitness of a Cry1Ac‐resistant strain of *Helicoverpa armigera* on Bollgard I plants (Bird and Akhurst, 2004, 2005). In the Cry1Ac‐resistant *H. armigera* strain, the intrinsic rate of increase *r* was reduced >50% with a significant decrease in survival rate and a 7‐day delay of development when grown on Bollgard I plants (Bird and Akhurst, 2004, 2005). As the genetic mechanism of the Cry1Ac resistance in that *H. armigera* strain is not known, whether the difference between *T. ni* and *H. armigera* in reduction in fitness by the toxin Cry1Ac in cotton plants is due to different resistance mechanisms or due to intrinsic physiological and metabolic difference between the two species is unknown.

Neither the GLEN‐Cry1Ac‐BCS strain nor the GLEN‐Cry2Ab‐BCS strain could survive on Bollgard II leaves, which was expected as there is no significant cross‐resistance between the Cry1Ac and Cry2Ab resistance in *T. ni* (Kain *et al*., 2015; Song *et al*., 2015; Wang *et al*., 2007). The presence of the dual toxins, Cry1Ac and Cry2Ab, in cotton plants more greatly reduced the fitness of the *T. ni* strain GLEN‐Cry1Ac+Cry2Ab‐BCS when feeding on Bollgard II leaves. The size of GLEN‐Cry1Ac+Cry2Ab‐BCS population could grow on Bollgard II leaves but exhibited an extended larval developmental time and a reduced survival rate. The further reduction in fitness of the GLEN‐Cry1Ac+Cry2Ab‐BCS strain on Bollgard II leaves can be attributed to both the fitness cost associated with the Cry1Ac resistance and Cry2Ab resistance (Wang *et al*., 2016) and also the low toxicity of the two toxins to the GLEN‐Cry1Ac+Cry2Ab‐BCS strain (Kain *et al*., 2015; Song *et al*., 2015; Wang *et al*., 2007).

The negative effect of the toxins in Bollgard II leaves on *T. ni* was mainly in the larval stage, shown as reduced survival and slower development, as larval stage is the developmental stage in which the insects are directly exposed to the Bt toxins and plant chemicals. However, the slower larval development on Bollgard II leaves did not lead to a significant change in pupal weight, emergence of healthy adults and the sex ratio. The 1‐day delay of the peak of egg laying of GLEN‐Cry1Ac+Cry2Ab‐BCS strain grown on Bollgard II leaves, compared to the same strain on non‐Bt cotton leaves, was the only observed physiological consequence on the fecundity of adults from the larvae grown on Bollgard II leaves.

It is evident that the presence of Cry toxins in plants reduced the overall fitness of *T. ni* feeding on the plant leaves, exhibiting incomplete resistance. The GLEN‐Cry1Ac+Cry2Ab‐BCS strain showed a lower fitness than the GLEN‐Cry1Ac‐BCS strain when grown on non‐Bt cotton leaves (Wang *et al*., 2016). Interestingly, the relative fitness (i.e. the fitness on Bt plants relative to the fitness of the same strain on non‐Bt plants) of the two Cry1Ac‐resistant strains on Bollgard I leaves was similar, regardless the presence of only Cry1Ac resistance mechanism in the GLEN‐Cry1Ac‐BCS strain or the presence of the Cry1Ac resistance mechanism and an additional Cry2Ab resistance mechanism in the GLEN‐Cry1Ac+Cry2Ab‐BCS strain. Both *R*_0_ and *r* values for the resistant *T. ni* strains indicate that the resistant *T. ni* populations could survive and grow on the Bt plants. However, presence of a Bt toxin in plants reduces the fitness of the insects, and presence of multiple Bt toxins in plants more significantly reduces the fitness of the insects feeding on the plants. The results from this study showed that pyramiding of Bt toxins in plants not only decreases the probability for insects to develop resistance which requires multiple mechanisms of resistance to all toxins (Zhao *et al*., 2003), but also reduces the fitness of resistant insects on the Bt plants to decrease their population growth, further delaying the selection of resistance in the insect populations (Wei *et al*., 2015). With the observations on the decline of relative fitness of resistant insects on Bt plants, it is expected that with more insecticidal genes stacked in a transgenic plant, the relative fitness of resistant *T. ni* on multitoxin plants will be further decreasing.

An asynchrony between adult stages of susceptible and resistant insects has been reported when the insects were grown on secondary host plants or on plants expressing a single Cry toxin (Bird and Akhurst, 2004; Gryspeirt and Gregoire, 2012; Wang *et al*., 2016). In this study, the developmental delay in the GLEN‐Cry1Ac+Cry2Ab‐BCS on Bollgard I leaves was observed to be 1.5 days. The GLEN‐Cry1Ac+Cry2Ab‐BCS strain could survive on Bollgard II leaves but had a much slower larval development, which resulted in a 12.4‐day asynchrony between the adults of the dual‐toxin‐resistant strain grown on Bollgard II leaves and the susceptible adults on non‐Bt cotton leaves. The refuge strategy relies on an assumption that the mating of insects in the field is panmictic among the adults from susceptible and resistant populations (Gould, 1998; Tabashnik, 2008). However, with the 12.4‐day developmental delay, the active adult stage of the resistant *T. ni* from the Bt cotton leaves will be out of phase with the active susceptible adults from non‐Bt cotton leaves. In *T. ni*, females generally mate within the first 3–5 days after emergence (Shorey, 1964). Therefore, a >12‐day asynchrony between the susceptible and resistant populations could potentially lead to temporal separation of the susceptible and resistant populations, resulting in resistant adults predominantly mating within the resistant population, rather than random mating between the resistant and susceptible individuals. Such assortative mating can reduce the efficacy of refuge strategy for delaying resistance development (Liu *et al*., 1999; Peck *et al*., 1999).

The series of studies on the fitness cost associated with resistance to Bt toxins Cry1Ac and Cry2Ab in *T. ni* on various host plants (Wang *et al*., 2016) and the effects of the Bt toxins in transgenic cotton plants on the fitness of *T. ni* described above indicate that selection of Bt resistance in insect populations can be affected by multiple factors related to fitness of the insects in the field. First of all, successful resistance of insects to Bt plants relies on the Bt resistance mechanisms that confer a sufficiently high level of resistance to the Bt toxins. However, resistance‐conferring gene mutations are often associated with a fitness cost and the fitness cost in an insect is affected by the host plants on which the insect is grown (Carriere *et al*., 2005). The fitness is generally higher when the insect is grown on a well‐adapted preferred host plant, but is lower on alternative host plants (Janmaat and Myers, 2005, 2007; Wang *et al*., 2016). The Bt toxins in Bt plants will further impact the fitness of the resistant insects when feeding on Bt plants. Furthermore, the results from this study on Bt resistance in *T. ni* also demonstrated that development of Bt resistance in an insect may be associated with not only a reduced population growth rate, but also slower larval development, leading to asynchrony of the susceptible and resistant populations. Such an asynchrony between the susceptible and resistant populations could be more profound when the resistant populations are grown on Bt plants. Therefore, development of insect resistance to Bt toxins in the field is complex. The fitness of resistant insects in the field depends on various factors including the host plants that the insects feed on, the mechanisms of resistance and the Bt toxins produced in the plants.

## Experimental procedure

### Insect strains

Four *T. ni* strains were used to examine their performance on foliage of Bt and non‐Bt cotton plants. The Cornell strain (Wang *et al*., 2007), a highly inbred laboratory strain, was used as the Bt‐susceptible control strain. Bt‐resistant strains used in this study included a Cry1Ac‐resistant strain, GLEN‐Cry1Ac‐BCS (Wang *et al*., 2007), a Cry2Ab‐resistant strain, GLEN‐Cry2Ab‐BCS (Song *et al*., 2015), and a strain resistant to both Cry1Ac and Cry2Ab, GLEN‐Cry1Ac+Cry2Ab‐BCS (Kain *et al*., 2015). These three resistant strains were near‐isogenic to the Cornell strain by introgression of the Cry resistance traits into the Cornell strain (Song *et al*., 2015; Wang *et al*., 2007). All *T. ni* strains were maintained on artificial diet without exposure to Bt toxins as previously described (Kain *et al*., 2004).

### Plants

Cotton plants of the non‐Bt cotton variety Stoneville 474 and the Cry1Ac‐producing cotton Bollgard I (event 531) and the dual‐toxin (Cry1Ac + Cry2Ab)‐producing cotton Bollgard II (event 15 985) were prepared as previously described by Kain *et al*. (2015). All plants were grown in formulated potting soil, the Cornell Mix (Boodley and Sheldrake, 1982), in 6‐L plastic pots in the glasshouse. Eight‐ to ten‐week‐old plants were used in this study.

### Examination of fitness costs

To record the development and mortality of *T. ni* of the susceptible and resistant strains, the larvae were reared on detached cotton leaves in 8.0 cm × 6.5 cm (D × H) paper cups. Ten neonates from each strain were placed in a cup, and 30 cups were used for each strain (300 larvae in total per *T. ni* strain). The cups were kept in an insect rearing room at 25 ± 1 °C, 50 ± 10% relative humidity and 16:8‐h photoperiod. Cotton leaves were replaced every 48 h before the larvae reached 4th instar and were replaced every 24 h for larvae in 4th instar. When the larvae were in 5th instar, cotton leaves were replaced daily or more frequently as necessary. Larval development and mortality were recorded in an interval of 12 h till all larvae were pupated. Pupae were collected daily, and their weights were measured and their sex was visually determined as previously described (Wang *et al*., 2016).

For examination of fecundity, one female adult and two male adults from the same strain reared on the same cotton variety were placed in a metal wire cage (ca. 12 cm in diameter and 11 cm in height) and were provided with 10% sugar solution. Thirty ‘1‐female + 2‐males’ cages were used as replications for each treatment. The cage was wrapped with wax paper for egg collection, and the wax paper was replaced daily. Eggs on the wax paper were counted, and egg hatching was examined daily afterwards.

The net reproductive rate (*R*_0_) and the intrinsic rate of increase (*r*) were calculated for each *T. ni* strain on different cotton varieties using the formula described by Carey (1993). For each of the 30 biological replicates from each condition (strain × cotton plant), *R*_0_ was calculated as *R*_0_ = *N*_n+1_/*N*_n_, where *N*_n_ is the population size of the parent generation and *N*_n+1_ is that of its next generation. For *R*_0_ calculation, the mean sex ratio was applied to each replicate to calculate the number of females from the next generation, while all other parameters (total number of adults, number of eggs laid and percentage of hatching) were measured data for each replicate. *R*_0_ > 1 indicates that the number of offspring females is greater than that of the parental females. *r* was calculated as *r *= ln(*R*_0_)/((*x* * *l*_*x*_ * *m*_*x*_)/*R*_0_), where *x* is the females age in days, *l*_*x*_ the age‐specific survival, *m*_*x*_ the age‐specific fecundity and *R*_0_ the net reproductive rate. *r *>* *0 indicates that the population size is increased, while *r *<* *0 indicates collapsing of the population. The relative fitness (*w*) of the resistant strains on transgenic plants was calculated as the ratio of *R*_0_ or *r* of the strain on transgenic cotton to *R*_0_ or *r* of the same strain on non‐Bt cotton foliage.

### Statistical analysis

The individual cups were treated as independent units in the analysis, using the mean of data from 10 larvae in each cup or from a ‘1‐female + 2‐males’ for each mating group. A Shapiro–Wilk test was performed for verifying the normality of the data. An ANOVA followed by multiple pairwise comparisons of means (Tukey's HSD test) was performed to test the statistical significance of the differences in observed life parameters measured between resistant and susceptible strains on the same plant or for the same strains between transgenic and conventional cottons. Pairwise comparisons of egg‐laying kinetics were performed with a two‐sample two‐sided Kolmogorov–Smirnov test. All statistical analyses were performed using the software R 3.0.2 (R Development Core Team, 2011).

## Conflict of interest

The authors declare no competing financial interests.

## Author Contributions

R.W. and P.W. designed the experiments. R.W. performed the experiments. G.T. performed the statistical analyses. G.T. and P.W. analysed the data and wrote the article. All authors contributed to manuscript revision and editing.
